# Evaluation of glycemic response and starch digestibility in Korean rice toward the development of low GI rice

**DOI:** 10.3389/fpls.2025.1724565

**Published:** 2025-12-11

**Authors:** Chang-Min Lee, O-Young Jeong, Hyun-Su Park, Jeonghwan Seo, Songhee Park, Keon-Mi Lee, Mina Jin, Il-Ryong Choi, Jae-Hyuk Han

**Affiliations:** 1 Crop Breeding Division, National Institute of Crop and Food Science, Rural Development Administration, Wanju, Republic of Korea; 2 IRRI-Korea Office, The International Rice Research Institute, Wanju, Republic of Korea

**Keywords:** diabetes, rice, glycemic index, starch, amylose, protein

## Abstract

The global rise in type 2 diabetes has intensified the need for dietary strategies that promote glycemic control, including the development of functional staple foods with a low glycemic index (GI). In this study, we assessed 16 Korean rice accessions for grain morphology, biochemical composition, starch digestibility, and glycemic response to identify promising candidates for low GI rice breeding. Substantial variation was observed in amylose (4.3–41.8%) and protein content (6.09–9.28%), both key factors influencing starch hydrolysis and postprandial glucose levels. *In vitro* GI assays showed that seven accessions had GI values below 60, including Dodamssal, Goami2, and Goami4, which were characterized by high resistant starch (RS) content. Interestingly, Seullomi1 and Seullomi2 maintained low-to-intermediate GI values, associated with elevated proportions of slowly digestible starch (SDS), moderate-to-high amylose content resistance to enzymatic digestion. Starch digestibility profiling confirmed that these lines retained higher SDS content even after cooking, suggesting greater resistance to enzymatic hydrolysis. *In vivo* murine study (*n* = 5 per group) further validated these findings, with Seullomi1, Seullomi2, and Dodamssal showing significantly reduced blood glucose spikes at 30 minutes compared with Sindongjin, a commercial Korean rice variety. Complementary textural analysis revealed that the Seullomi lines preserved moderate hardness and stickiness, traits favorable for consumer acceptance. Collectively, these results establish Seullomi1 and Seullomi2 as promising prototypes for the development of functional rice varieties that provide glycemic control with acceptable textural quality, thereby facilitating the breeding of rice to mitigate the risk of type 2 diabetes.

## 1 Introduction

The prevalence of type 2 diabetes is rapidly increasing worldwide, driven largely by dietary patterns high in refined carbohydrates and foods with a high glycemic index (GI). This global trend highlights the urgent need for dietary interventions targeting staple foods to mitigate the health risks associated with diabetes. Healthcare costs related to diabetes reached approximately US$966 billion and are expected to exceed US$1,054 billion by 2045. This increase is closely associated with a shift in dietary patterns toward energy-dense foods, such as sugar-sweetened beverages, ultra-processed items, and refined carbohydrates (Ogurtsova et al., 2022; Sun et al., 2022). Consequently, there is a growing consumer demand for functional foods that support glycemic control while maintaining favorable sensory and nutritional qualities (Collar and Angioloni, 2014).

Rice (*Oryza sativa* L.), one of the three major global cereal crops, is a staple food for more than half of the world’s population and contributes up to 40% of daily caloric intake in many Asian countries. It is gluten-free, easily digestible, low in sodium, hypoallergenic, and contains essential nutrients such as vitamins B and E, phosphorus, and folic acid (Choi, 2012; Um and Yoo, 2013). However, despite these benefits, high-quality rice varieties typically have GI values ranging from 70 to 92, classifying them as high GI foods (Jukanti et al., 2020). High GI rice is rapidly digested and absorbed, leading to sharp increases in postprandial blood glucose levels and, consequently, an elevated risk of type 2 diabetes. In response to emerging dietary needs, breeding programs in countries such as China, India, the Philippines, and Australia have prioritized the development and commercialization of low GI rice cultivars (Cabral et al., 2022; Tiozon et al., 2024).

Recent advancements in rice breeding techniques have enabled the targeted development of low GI rice through marker-assisted selection, mutagenesis, and genome editing. These approaches focus on modifying starch composition by increasing amylose content (AC) and resistant starch (RS) to reduce starch digestibility and glycemic response. Genome-editing tools, such as CRISPR/Cas9, have enabled the precise modification of starch biosynthetic genes, including *SBEIIb* and *SSIIIa*, resulting in rice lines with significantly lower glycemic potential. Excessive levels of AC or RS can result in a more complex texture or less desirable eating quality. Therefore, it is essential to balance the reduction of postprandial blood glucose levels with the maintenance of grain yield, consumer-preferred texture, and the enhancement of nutritional traits (Tiozon et al., 2024).

To address this challenge, Badoni et al. (2024) developed recombinant inbred lines (RILs) by crossing Samba Mahsuri with the *IR36 amylose extender* (*IR36ae*) mutant. Utilizing a multi-omics approach that included QTL-seq, genotyping-by-sequencing, association mapping, and metabolomics, they identified key loci controlling glycemic index, amylose, and protein content. The gene *OsSBEIIb* was a crucial factor for low GI through CRISPR/Cas9. The high amylose and protein lines also exhibited enrichment in glycolytic and amino acid biosynthesis pathways, enhancing their functional value. Additionally, Chaichoompu et al. (2024) demonstrated that achieving a low to intermediate GI can be accomplished by selecting favorable ratios of soluble to insoluble dietary fiber (SDF/IDF), alongside optimizing amylose content, thereby securing both nutritional benefits and acceptable sensory attributes.

Starch contributes 75–80% of the weight of rice grains and plays a crucial role in determining glycemic response (Kim et al., 2022). It is classified into three types based on digestion kinetics: rapidly digestible starch (RDS), slowly digestible starch (SDS), and resistant starch (RS) (Englyst et al., 1992). RDS is broken down within 20 minutes and is strongly linked to a high GI and rapid glucose release (Hsu et al., 2015; Miller et al., 1992). In contrast, SDS is digested throughout 20 to 120 minutes and helps maintain sustained glycemic control. RS, on the other hand, is not digested in the small intestine; instead, it is fermented in the colon, providing benefits similar to dietary fiber (Birt et al., 2013). A higher proportion of SDS and RS has been shown to lower GI and improve metabolic health outcomes (Magallanes-Cruz et al., 2017).

RS reduces glycemic response, lowers cholesterol levels, and promotes gut health. In rice, the levels of RS are affected by various factors, including the architecture of starch granules, crystal structure, the amylose-to-amylopectin ratio, and their interactions with lipids and proteins. Key genes such as *GBSSI (Wx)*, *SSIIa*, *SSIIIa*, and *SBEIIb* regulate RS biosynthesis and branching patterns. Genetic modifications targeting these genes have successfully increased RS content, although this often comes at the expense of grain yield and quality. Current breeding strategies are focused on fine-tuning gene expression and employing genome-editing techniques to optimize RS levels without compromising agronomic performance (Shen et al., 2022).

Similarly, SDS is characterized by its gradual enzymatic breakdown in the small intestine, resulting in a moderate release of glucose and prolonged feelings of fullness. The formation of SDS is closely linked to the structure of starch granules, including semi-crystalline regions, the branching of amylopectin, and the type of crystal present (Lehmann and Robin, 2007). Various processing techniques, such as annealing and heat-moisture treatment (HMT), can increase the SDS content by reinforcing the order of the starch and making it less susceptible to enzymatic breakdown. Enhancing SDS content through physicochemical and genetic methods in rice breeding is a promising approach to achieving low GI properties while maintaining grain quality (da Rosa Zavareze and Dias, 2011). Therefore, it is important to study the combination of genetic, molecular, and processing-based strategies to control starch digestibility and identify germplasms for low GI rice.

To our knowledge, none of the 16 rice accessions have been systematically evaluated for their *in vitro* and *in vivo* glycemic responses. While low GI rice varieties have been reported in other regions, the potential of Korean germplasm for low GI rice remains underexplored. This study aims to characterize the morphological, biochemical, and glycemic properties of 16 Korean rice accessions to identify promising candidates for developing low-GI rice with acceptable textural properties.

## 2 Materials and methods

### 2.1 Plant materials and grain quality characterization

A total of 16 rice accessions, comprising nine *japonica* and seven *indica* types developed by the National Institute of Crop Science (Republic of Korea), were used in this study (**Table 1**). Grain length and width were measured using a digital caliper (CD-15CP, Mitutoyo Corp., Kawasaki, Japan), and the length-to-width ratio was calculated. Protein content was quantified using the AOAC (2000) method via an automated Kjeldahl-based protein analyzer (Kjeltec 2400 AUT, Foss, Mulgrave, Australia). The amylose content was analyzed using the colorimetric method outlined by Juliano (1985). Initially, 100 mg of rice flour was treated with 95% ethanol and 1 N sodium hydroxide. The resulting gelatinized starch suspension was then reacted with 1 N acetic acid and a 2% I_2_-KI solution. Finally, the absorbance was measured at 620 nm using a spectrophotometer to determine the amylose concentration.

**Table 1:** Physical and biochemical characterization of 16 rice accessions.

Entry no.	Sample	Cross combination	Brown rice				Amylose content (%)	Protein content (%)
			Length (mm)	Width (mm)	L/W ratio	Thickness (mm)		
IKO23001	Nampyeong^a^	Iri390/Milyang95	4.81	2.93	1.65	2.01	19.56 ± 0.05	6.65 ± 0.06
IKO23002	Milyang 385^a^	Hopum/YR22156-B-B-B-4	5.08	3.05	1.67	2.12	17.93 ± 0.24	6.79 ± 0.07
IKO23004	Sindongjin^a^	Hwayeongbyeo/YR13604Acp22	5.83	3.03	1.92	2.07	19.43 ± 0.21	6.19 ± 0.01
IKO23005	Sinseonchal^a^	Milyang20/Hiyokumochi	5.19	2.90	1.79	2.03	4.30 ± 0.08	6.74 ± 0.05
IKO23006	Hanyeol^b^	Senpidao/Huan Hua Zhan	6.53	2.15	3.04	1.74	18.65 ± 0.10	7.11 ± 0.05
IKO23007	Seullomi1^b^	OM5930/Dular//OM5930///AS996	7.49	2.22	3.39	1.82	25.99 ± 0.23	7.95 ± 0.04
IKO23008	Seullomi3^b^	OM5930/Dular//CKR1-240-1/Chulsa	7.18	2.14	3.36	1.74	24.70 ± 0.13	7.37 ± 0.04
IKO23009	Seullomi2^b^	OM5930/Dular//CKR1-240-1/Chulsa	6.55	2.05	3.20	1.74	20.51 ± 0.36	9.28 ± 0.02
IKO23010	KR1533-50-1-1-2-2^b^	Pusa Basmati 1//CKR1-240-1/Chulsa	6.94	2.27	3.06	1.79	19.49 ± 0.12	6.66 ± 0.05
IKO23011	KR2246-11-1^b^	Senkra Ob/Taebaeg	7.28	2.39	3.05	1.95	20.98 ± 0.22	7.09 ± 0.03
IKO23012	Seullomihyang^b^	Senkra Ob/Taebaeg	6.90	2.70	2.56	1.98	21.22 ± 0.19	6.75 ± 0.06
IKO23015	Goami^a^	Milyang95//Kimcheonangmi/Ilpumbyeo*2	5.35	2.97	1.80	1.96	27.91 ± 0.23	7.09 ± 0.02
IKO23016	Goami2^a^	Ilpumbyeo MNU	4.78	2.87	1.67	1.87	32.64 ± 0.26	6.27 ± 0.08
IKO23020	Goami4^a^	Suweon464/Daeanbyeo	5.19	2.97	1.75	1.78	34.45 ± 0.23	7.34 ± 0.05
IKO23022	Dodamssal^a^	Goami/Goami2	5.17	3.09	1.68	2.09	41.83 ± 0.25	6.09 ± 0.04
IKO23023	Baekjinju^a^	Ilpumbyeo MNU	4.77	2.89	1.65	2.05	9.99 ± 0.17	6.33 ± 0.03

a Oryza sativa subsp. japonica.

b Oryza sativa subsp. indica.

### 2.2 *In vitro* glycemic index assay

The *in vitro* glycemic index (GI) was evaluated following the protocol standardized by the International Rice Research Institute (IRRI) (Pautong et al., 2022). 300 ± 0.3 mg of milled rice was cooked at a 1:2 rice-to-water ratio for 23 minutes in boiling water, cooled to room temperature for 5 minutes, and equilibrated at 37°C. One milliliter of 0.1 M sodium-potassium phosphate buffer (pH 6.9) was added, and the sample was gently mashed using a stainless-steel spatula. Then, 2 mL of α-amylase (55.5 U/mL, Sigma) was added and incubated at 37°C for 75 seconds before quenching the reaction with 3 mL of HCl (pH ~1.0). Pepsin (19.5 U/mL, Sigma) was added and incubated at 37°C for 30 minutes. The pH was adjusted to 6.9 using 15 mL of NaOH (pH ~12.6). At 0 and 30 minutes, samples were collected and stored at 4°C. Digestion was continued with a 15 mL enzyme mixture containing pancreatin (28.4 μg/mL) and amyloglucosidase (13 U/mL) in 0.1 M phosphate buffer (pH 6.9) at 37°C. Glucose concentrations at 0 and 30 minutes were determined using the GOPOD assay (Megazyme D-Glucose Assay Kit) at 510 nm. The hydrolysis area under the curve (AUC) was calculated and converted to GI using the regression equation: pGI = 0.0781 × AUC_corrected + 47.8876.

### 2.3 *In vitro* starch digestibility

Starch digestibility was measured according to the modified Englyst et al. (1992) method, as adapted by Mansur et al. (2022). A mixture of porcine pancreatin and amyloglucosidase was prepared by stirring 2 g of pancreatin in 24 mL of distilled water for 10 minutes, followed by centrifugation at 1500×g for 10 minutes at 4°C. The supernatant was mixed with 0.4 mL of amyloglucosidase and 3.6 mL of distilled water. For each sample, 30 mg of rice flour was combined with 0.75 mL of 0.1 M sodium acetate buffer (pH 5.2) and a glass bead in a microtube, followed by pre- and post-cooking. After cooling to 37°C, 0.75 mL of the enzyme solution was added, and the mixture was incubated at 240 rpm. Samples were withdrawn at 20 and 240 minutes, boiled to stop enzymatic activity, cooled to 20–23°C, and centrifuged at 5000×g for 10 minutes. The supernatant was analyzed for glucose using a GOD-POD assay. Glucose released at 20 minutes represented rapidly digestible starch (RDS), at 20–240 minutes represented slowly digestible starch (SDS), and the remaining fraction was classified as resistant starch (RS).

### 2.4 Structural characteristics analysis of starches

X-ray diffraction (XRD) was conducted using a powder diffractometer (New D8 Advance, Bruker, Karlsruhe, Germany) with Cu-Kα radiation (λ = 1.54 Å) at 40 kV and 40 mA. Scans were recorded from 3° to 40° (2θ) with a step size of 0.02°. Relative crystallinity was calculated using Origin software (v7.5, OriginLab, Northampton, MA, USA) with the formula: Relative Crystallinity (%) = Ac/(Ac + Aa) × 100, where Ac is the area under crystalline peaks, and Aa represents the amorphous region.

### 2.5 Texture characteristics analysis of cooked rice

The textural properties were measured using the Tensipresser (My Boy II System, Taketomo Electric Inc., Tokyo, Japan), a device developed in Japan as a convenient method for mechanically measuring the chewiness that people sensually perceive when eating rice (Kim et al., 2018). A sample of 30 g of white rice was placed in a stainless-steel cup (height, 8 cm; diameter, 4 cm) and lightly stirred. It was then rinsed with water twice before soaking for 4 hours. The soaked sample was placed in a self-made multi-point cooker, heated on high for 10 minutes, then cooked on medium heat for 10 minutes, and finally on low heat for 10 minutes. After removing the water from the container, it was allowed to rest on low heat for about 10 minutes, and then the sample was left to cool to room temperature. The cooked rice was randomly weighed in 10 g portions and formed into a sample cup, rested for 2 minutes, and then attached to the Tensipresser equipped with a puncture probe (contact area 25 mm²) for a measurement under a load of 20 Kgw, repeating the pressure test for the first bite at 25% and the second bite at 90%, five times. The investigation measured the hardness, adhesiveness, toughness, and stickiness of the rice (Takahashi et al., 2000).

### 2.6 *In vivo* glycemic response in mice

Animal experiments were conducted based on the protocol of Mansur et al. (2022) and approved by the Institutional Animal Care and Use Committee (IACUC) of Wonkwang University (Approval No. WKU24-21). Fifty female ICR mice (4 weeks old, 20–22 g) were acclimated for 7 days under standard laboratory conditions (23 ± 3°C, 60 ± 10% humidity, 12 h light/dark cycle) and divided into ten groups (*n* = 5). After fasting for 12 hours, mice were orally administered 0.5 mL of test rice suspension (7.5%, w/v) or glucose solution (control) using an oral Zonde needle. Blood samples were collected from the tail vein at 0, 30, 60, 90, 120, 150, 180, and 240 minutes. Blood glucose levels were measured using an Accu-Chek Instant Meter (Roche, Basel, Switzerland). Glycemic responses were calculated as the area under the glucose response curve (AUC), expressed as a percentage of the control.

### 2.7 Statistical analysis

All experiments were carried out in triplicate unless stated otherwise. The results are presented as means ± standard deviation (SD). A one-way analysis of variance (ANOVA) was conducted using SPSS software (version 23.0; SPSS Inc., Chicago, IL, USA), and significant differences among the means were assessed using Duncan’s multiple range test with a confidence level set at *P* < 0.05.

## 3 Results

### 3.1 Morphological and biochemical characteristics of 16 Korean rice varieties

A comprehensive evaluation of the grain morphology and biochemical profiles of 16 Korean rice accessions, comprising nine *japonica* and seven *indica* (**Table 1**). The length-to-width ratio, used to determine grain shape, ranged from 1.65 for round and bold grains (e.g., Nampyeong and Baekjinju) to 3.39 for long and slender grains (e.g., Seullomi1 and Seullomi2). Amylose content varied significantly among the accessions, with Sinseonchal having the lowest level at 4.3%, indicating that it is glutinous rice. In contrast, Dodamssal exhibited the highest amylose content at 41.8%, which is characteristic of non-sticky, harder-textured rice. Other accessions, such as Goami2 (32.6%) and Goami4 (34.4%), also showed high amylose levels. Protein content ranged from 6.09% (Dodamssal) to 9.28% (Seullomi2), underscoring substantial genotypic variability in grain nutritional composition (**Table 1**).

### 3.2 *In vitro* glycemic index assessment

The glycemic index (GI) of each rice accession was evaluated through *in vitro* evaluation, including glycemic index, digestible carbohydrate (%DC), and resistant starch (%RS) (**Table 2**). Goami4 (4.30%), Goami2 (4.11%), and Dodamssal (3.51%) exhibited high levels of resistant starch, suggesting slower digestion and reduced glycemic responses. In contrast, Sinseonchal (0.14%) and Sindongjin (0.20%) showed low levels of resistant starch, indicating rapid digestibility. The GI values varied, with Dodamssal at 52.52 classified as low GI, while Baekjinju at 87.76 was classified as high GI. Notably, Dodamssal and Seullomi3 demonstrated the most favorable glycemic index levels. At the same time, Sinseonchal and Baekjinju were associated with rapid glucose release and may be less suitable for individuals managing postprandial glucose levels.

**Table 2:** Evaluation of *in vitro* glycemic index assay.

Entry no.	%DC^a^	%RS^b^	GI^c^	Type^d^
Nampyeong	91.14 ± 0.81	0.25 ± 0.06	76.35 ± 4.97	HIGH GI
Milyang385	91.43 ± 2.23	0.29 ± 0.02	69.94 ± 0.26	INT GI
Sindongjin	96.27 ± 2.67	0.20 ± 0.00	76.85 ± 2.82	HIGH GI
Sinseonchal	86.73 ± 2.01	0.14 ± 0.03	87.66 ± 10.36	HIGH GI
Hanyeol	93.65 ± 5.55	0.26 ± 0.05	75.97 ± 5.58	HIGH GI
Seullomi1	88.23 ± 8.40	0.35 ± 0.05	55.37 ± 1.94	INT GI
Seullomi3	84.80 ± 0.15	0.37 ± 0.09	54.69 ± 3.26	LOW GI
Seullomi2	84.66 ± 2.97	0.29 ± 0.06	59.84 ± 3.00	INT GI
IKO23010	93.65 ± 0.05	0.33 ± 0.18	69.17 ± 8.03	INT GI
IKO23011	91.92 ± 2.40	0.19 ± 0.00	66.05 ± 1.72	INT GI
Seullomihyang	90.36 ± 0.91	0.23 ± 0.00	63.50 ± 6.46	INT GI
Goami	91.82 ± 2.55	0.50 ± 0.03	57.04 ± 1.25	INT GI
Goami2	81.08 ± 1.30	4.11 ± 0.91	58.17 ± 1.76	INT GI
Goami4	72.60 ± 0.85	4.30 ± 1.08	58.98 ± 3.67	INT GI
Dodamssal	89.34 ± 0.61	3.51 ± 0.96	52.52 ± 0.05	LOW GI
Baekjinju	87.27 ± 1.65	0.18 ± 0.00	87.76 ± 8.86	HIGH GI

a DC, Digestible carbohydrate (%).

b RS, Resistant starch (%).

c GI, Glycemic index.

d LOW GI (55 or less), INT GI (56-69), HIGH GI (70 or above).

### 3.3 *In vitro* starch digestibility analysis

To gain a comprehensive understanding of the relationship between starch properties and glycemic index, an evaluation was conducted on eight rice accessions, focusing on their *in vitro* starch digestibility profiles. The samples were analyzed for their contents of rapidly digestible starch (RDS) and slowly digestible starch (SDS) in both pre- and post-cooking rice flour (**Table 3**). In raw flour, RDS levels ranged from 36.3% (Dodamssal) to 52.2% (Hanyeol), while SDS levels varied from 25.3% (IKO23010) to 36.5% (Seullomi1), indicating a higher sustained glucose release. Upon cooking, RDS fractions rose markedly (67.9–76.0%), while SDS fractions declined sharply in most accessions. Notably, Seullomi1 (7.69%) and Seullomi2 (8.14%) showed significant SDS content, indicating considerable structural resistance to gelatinization.

**Table 3:** Digestible starch analyses in rice flour.

Sample	Pre-cooking		Post-cooking	
	RDS (%)	SDS (%)	RDS (%)	SDS (%)
Sindongjin	50.1 ± 1.64^d^	29.5 ± 1.75^b^	76.0 ± 1.52^c^	2.76 ± 1.84^ab^
Hanyeol	52.2 ± 0.45^d^	26.3 ± 0.72^a^	74.3 ± 0.70^c^	2.84 ± 0.61^ab^
Seullomi1	40.1 ± 1.37^b^	34.6 ± 1.34^c^	69.8 ± 1.05^ab^	7.69 ± 0.97^c^
Seullomi3	39.1 ± 1.72^b^	34.7 ± 1.42^c^	71.0 ± 1.98^b^	4.38 ± 1.58^b^
Seullomi2	38.1 ± 1.86^ab^	34.5 ± 1.61^c^	67.9 ± 0.53^a^	8.14 ± 1.45^c^
IKO23010	47.4 ± 1.35^c^	28.3 ± 0.78^ab^	73.5 ± 1.57^c^	1.95 ± 0.79^a^
Seullomihyang	50.1 ± 1.98^d^	28.3 ± 1.43^ab^	74.2 ± 0.73^c^	1.85 ± 1.66^a^
Dodamssal	36.3 ± 0.57^a^	29.6 ± 1.20^b^	69.2 ± 1.45^ab^	1.22 ± 0.10^a^

RDS, rapidly digestible starch; SDS, slowly digestible starch. Duncan’s multiple range test was used to compare means, and values within the same column followed by different letters are significantly different at *P* < 0.05.

### 3.4 Structural and textural properties analysis

**Figure 1:**
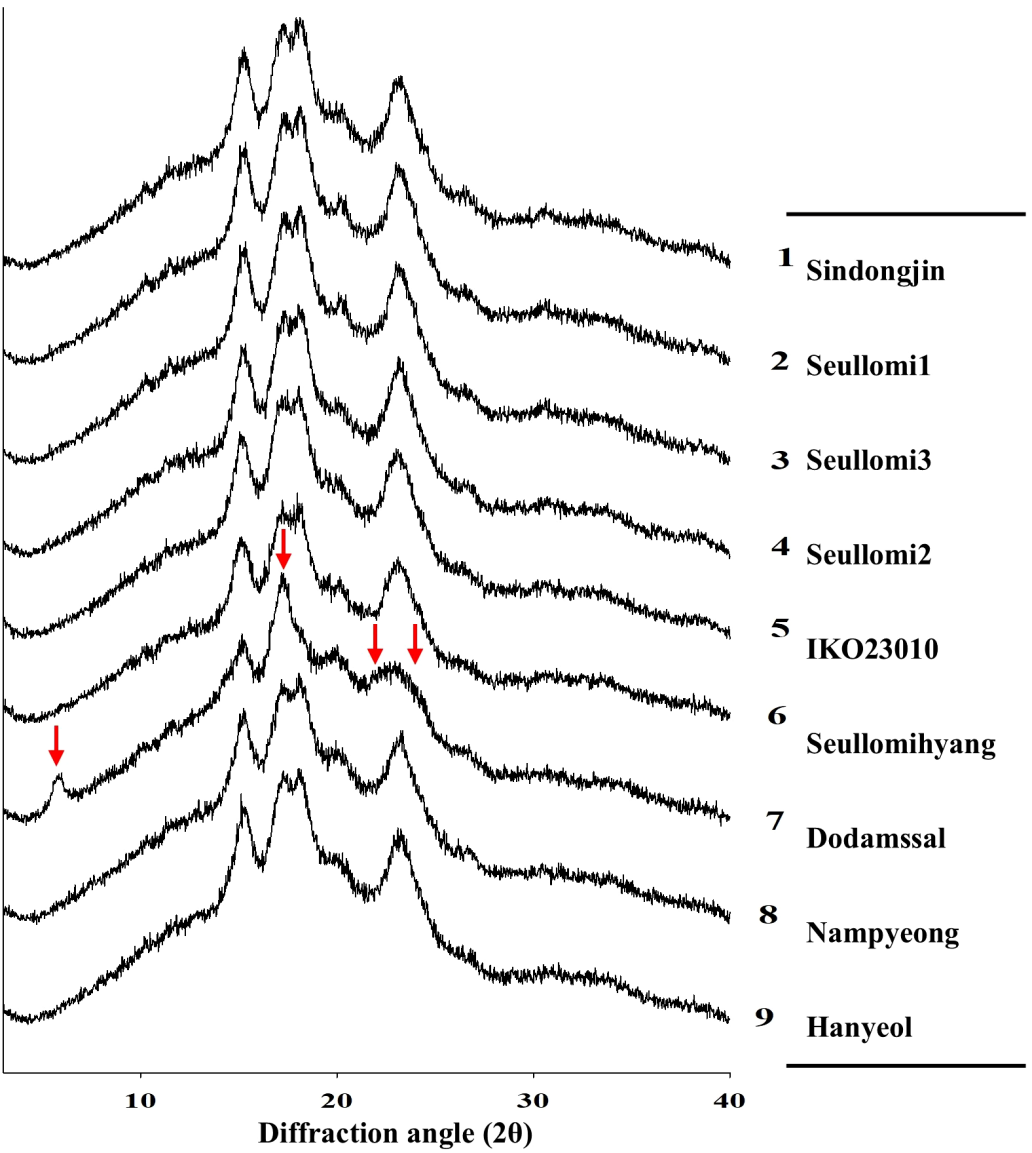
X-ray diffraction analysis. The red arrow indicates the strong peaks at diffraction angle (2θ) of 5.8°, 17°, 22°, and 24° in Dodamssal.

X-ray diffraction (XRD) was employed to examine the crystalline structures of nine rice accessions (**Figure 1**). Dodamssal exhibited a B-type crystalline pattern, characterized by distinctly strong peaks at diffraction angles (2θ) of 5.8°, 17°, 22°, and 24°. In contrast, the other accessions, including Sindongjin, Seullomi1, and Seullomi2, displayed a typical A-type pattern, marked by peaks at approximately 15°, 17-18°, and 23.4° 2θ. Grain cereals are categorized into A, B, and C types based on their starch composition. Generally, rice is classified as A-type, identified by distinct peaks at 15°, a shoulder peak between 17° and 18°, and a peak at 23.4° 2θ. The peak profile of Dodamssal closely resembles that of potato starch, which is characterized by a high RS content and is classified as B-type (Park et al., 2016). Following the RS content of Dodamssal is 3.51% after cooking (**Table 3**). Conversely, Seullomi1 and Seullomi2 are typical A-type rice, yet they exhibit high AC, low RS, and higher SDS levels, making them promising candidates for alternative low GI rice (**Tables 2**, **3**).

**Figure 2:**
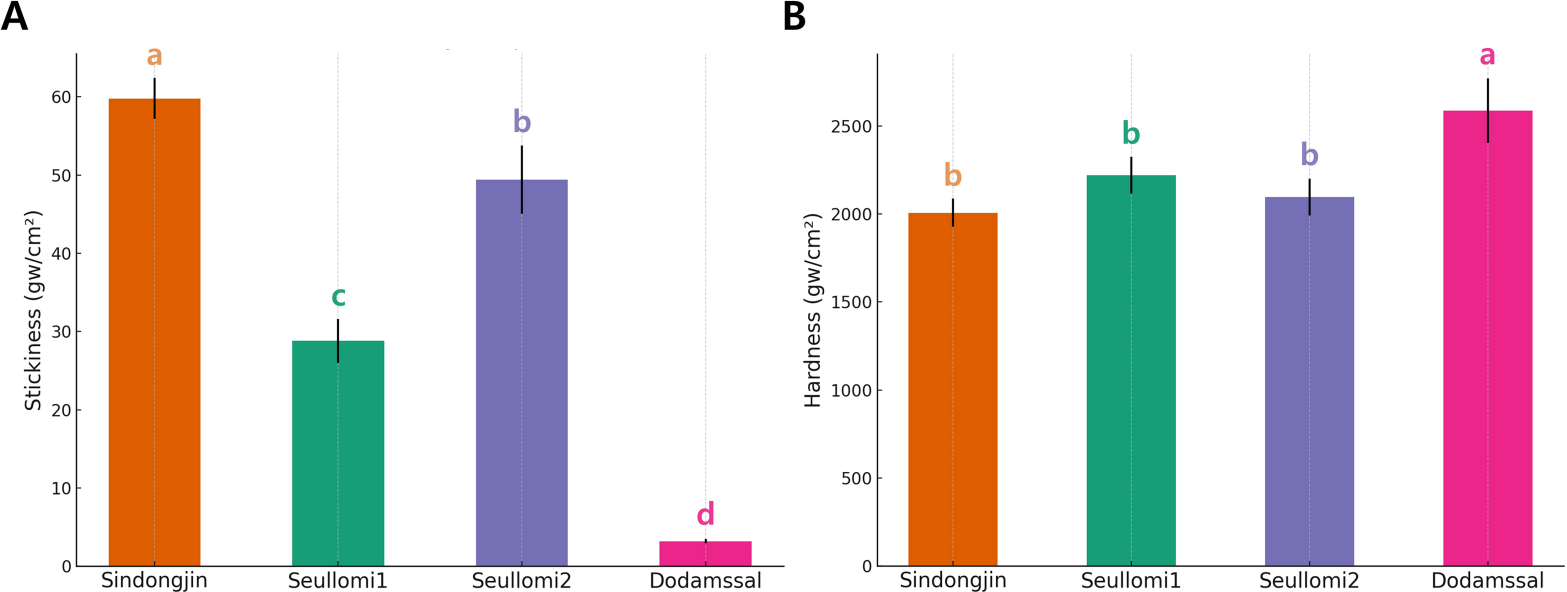
Textural properties analysis. Two textural attributes, stickiness **(A)** and hardness **(B)**, of four Korean rice accessions. Different letters indicate significantly different means at *P* < 0.05 according to Duncan’s multiple range test.

The textural properties of Seullomi1 and Seullomi2 were analyzed to determine a consumer-acceptable texture comparable to that of the Korean elite rice, Sindongjin (**Figure 2**). Following a standardized cooking protocol, the four accessions differed significantly in stickiness (**Figure 2A**). Sindongjin exhibited the greatest stickiness (60.2 ± 2.1 gW/cm²), significantly higher than Seullomi2 (49.6 ± 1.8 gW/cm²). Seullomi1 had an intermediate stickiness (29.1 ± 1.5 gW/cm²), exceeding Dodamssal but below Seullomi2. Dodamssal exhibited the lowest stickiness (3.2 ± 0.7 gW/cm²), which was significantly lower than that of all other accessions. These findings establish a clear hierarchy of stickiness among the variants examined. Sindongjin and Seullomi2 showed the highest stickiness, followed by Seullomi1, while Dodamssal showed the lowest stickiness. Hardness measurements (**Figure 2B**) revealed that Dodamssal was the firmest (2,620 ± 45 gW/cm²), while Sindongjin had the lowest hardness (2,010 ± 30 gW/cm²), a value statistically comparable to Seullomi1 and Seullomi2. Thus, Dodamssal’s cooked texture is markedly firmer, whereas Seullomi lines and Sindongjin share a similar, moderate hardness.

### 3.5 *In vivo* glucose response in murine models

**Figure 3:**
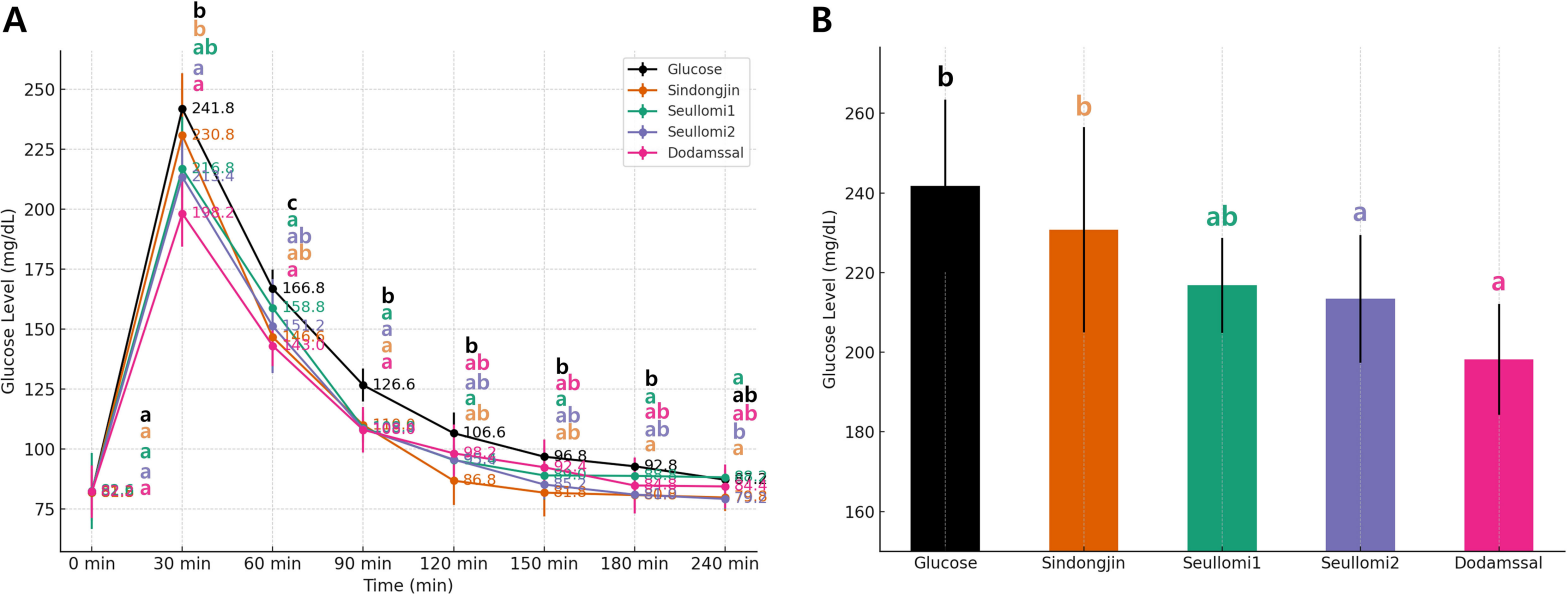
*In vivo* glucose response in murine models. **(A)** Blood glucose levels measured in mice fed different rice accessions. **(B)** Blood glucose levels measured in 30 min. Duncan’s multiple range test was used to compare means, and values with different letters indicate significant differences at *P* < 0.05.

*In vivo* glycemic responses were assessed in mice after oral administration of four rice samples and glucose control over 240 minutes (**Figure 3**). Glucose levels at the baseline were consistent across the groups, ranging from 81.8 to 82.2 mg/dL. At the 30-minute mark, the glucose showed the highest spike at 241.8 mg/dL, followed by the Sindongjin at 230.8 mg/dL, while Dodamssal exhibited the lowest increase at 198.2 mg/dL. By 60 minutes, glucose levels began to decline, with Dodamssal showing the lowest glucose level. This trend was observed consistently over 240 minutes, with Dodamssal exhibiting lower glucose levels compared to the other samples (**Figure 3A**).

However, the 30-minute postprandial glucose (30-min PG) level is considered a significant indicator for assessing the risk of developing type 2 diabetes (Hirakawa et al., 2020). This suggests that monitoring the 30-min PG level can provide valuable insights into an individual’s risk of developing type 2 diabetes. In our study, we found that Seullomi1, Seullomi2, and Dodamssal exhibited lower glucose levels compared to commercial rice varieties in Korea, such as Sindongjin (**Figure 3B**). These results suggest that Seullomi1, Seullomi2, and Dodamssal could serve as promising options for diabetes prevention.

## 4 Discussion

### 4.1 Addressing type 2 diabetes through the development of low GI rice in Korea

The rising global prevalence of type 2 diabetes is strongly associated with dietary patterns characterized by excessive consumption of rapidly digestible carbohydrates and high GI foods. This highlights the urgent need for nutritional interventions focusing on staple crops routinely consumed by large populations. International rice breeding programs have successfully developed low GI rice cultivars by targeting starch biosynthetic pathways, particularly through the enhancement of amylose content and modulation of resistant starch formation (Tiozon et al., 2024).

In Korea, although a wide range of genetic resources is available, systematic evaluation of starch digestibility components and *in vivo* glycemic responses has been limited. This study addresses this gap by integrating biochemical profiling, starch structural assessment, and murine glycemic response analysis to identify potential candidates for low GI rice breeding. Of particular importance is the identification of accessions that combine low to intermediate GI performance with acceptable texture for consumer preference. Accessions such as Seullomi1 and Seullomi2 demonstrate that achieving moderate GI is possible without excessively very high amylose, through a balanced combination of starch properties, including partial retention of SDS after cooking. While SDS may offer additional benefit in moderating digestive kinetics, its contribution should be considered complementary to the primary influence of amylose-mediated structural resistance. Therefore, the development of rice varieties that optimize glycemic control while maintaining favorable eating quality may support public health strategies to reduce the risk of diet-related metabolic disorders, including type 2 diabetes.

### 4.2 Biochemical mechanisms underlying glycemic response

Amylose content is a crucial factor influencing starch digestibility and postprandial glycemic response. High-amylose starch consists predominantly of linear α-1,4-linked glucose chains that form tightly packed double helices and crystalline structures, rendering it resistant to enzymatic degradation (Li, 2023). Consequently, high-amylose rice varieties exhibit lower starch gelatinization during cooking, leading to incomplete starch breakdown and reduced glucose release. Dodamssal, which has the highest amylose content among the tested accessions at 41.8%, exhibited a significantly lower GI value and a higher RS content, classified as resistant starch type 2 (RS2) (Li et al., 2024; Shen et al., 2022). RS2 originates from the native molecular arrangement of ungelatinized or partially gelatinized starch granules, which are known for their resistance to digestive enzyme action due to their compact structural configuration (Adeva et al., 2020; Park et al., 2023).

In contrast, Seullomi1 (26.0% amylose content, GI 55.37) and Seullomi2 (20.5% amylose content, GI 59.84) had low-to-intermediate GI values despite lower amylose levels than Dodamssal. This phenomenon can be attributed to the combined effects of moderate-to-high amylose content and SDS retention after cooking, which likely reduced the rate of enzymatic hydrolysis. Amylose-driven structural resistance is a primary regulator of starch digestibility, where linear chain interactions facilitate the formation of ordered crystalline networks that inhibit enzymatic access. These interactions also contribute to SDS formation, as reported by Doan et al. (2025), who demonstrated that amylose–amylose interactions reduce hydrolytic accessibility and increase both SDS and RS fractions. Therefore, SDS retention in Seullomi accessions should be considered a complementary effect to amylose-mediated resistance, rather than an independent mechanism.

The results collectively indicate that amylose-driven RS2 formation plays a pivotal role in glycemic regulation, while SDS serves as a complementary factor, particularly in genotypes exhibiting moderate amylose levels. The current experimental design limitations hinder the ability to isolate the effects of SDS from those associated with amylose-mediated structural characteristics. Consequently, the relationship between SDS and glycemic response should be interpreted as correlative rather than mechanistic. To validate the independent contribution of SDS, further studies utilizing genotypes with comparable amylose content but varying SDS levels are necessary.

### 4.3 Digestibility profiles and limitations in mechanistic interpretation

SDS plays a complementary role in moderating the postprandial glycemic response by gradually releasing glucose during digestion. In this study, Seullomi1, Seullomi2, and Seullomi3 exhibited higher SDS content in raw rice flour (34.6–34.7%) than in the commercial variety Sindongjin (29.5%), suggesting relatively structured starch granules with restricted enzymatic access. Following cooking, a substantial decrease in SDS was observed across all accessions due to gelatinization. However, Seullomi1 and Seullomi2 exhibited significantly higher SDS retention (7.69% and 8.14%, respectively) compared to other accessions, indicating partial resistance to cooking-induced starch disruption. This response is likely attributed to favorable starch molecular architecture, potentially involving shorter amylopectin chains and semi-crystalline lamellae, which restrict enzyme penetration.

Nevertheless, SDS activity must be interpreted within the context of additional starch properties, primarily amylose-mediated resistance. Since Seullomi1 and Seullomi2 possess moderate to high amylose content, the SDS-associated glycemic effects cannot be mechanistically separated from amylose-influenced starch regulation. Furthermore, we recognize that Seullomi2 exhibits slightly higher protein content, but this should be regarded as a minor compositional feature rather than a determinant of starch digestibility. Thus, while the elevated SDS observed in Seullomi accessions may be involved in moderating digestion, it should be regarded as correlated with, rather than directly responsible for, the observed glycemic outcomes. Future studies are needed to evaluate genotypes with equivalent amylose levels but contrasting SDS contents to determine whether SDS exhibits an independent regulatory role.

While we are proceeding cautiously with SDS, it is evident that Seullomi1 and Seullomi2 are promising low GI rice candidates. *In vivo* glycemic response demonstrated notable reductions in blood glucose levels at the crucial 30-minute post-meal time point for Seullomi1, Seullomi2, and Dodamssal, particularly compared with commercial Korean rice varieties such as Sindongjin (**Figure 3**). Given that elevated glucose levels at this interval are closely linked to a heightened risk of type 2 diabetes, these findings suggest that these accessions could play a significant role in diabetes prevention strategies.

### 4.4 Starch structural organization and textural properties

XRD analysis revealed structural differences among starches that were consistent with glycemic release (**Figure 1**). Dodamssal exhibited a B-type crystalline structure, typically associated with tighter molecular packing and limited enzymatic accessibility, suggesting the formation of RS2 in high-amylose starch. The high amylose content of Dodamssal, combined with incomplete gelatinization during cooking, supports the presence of RS2, contributing to its reduced digestibility and low GI. In contrast, Seullomi1 and Seullomi2 displayed A-type crystallinity, which is generally more susceptible to enzymatic hydrolysis and associated with higher levels of RDS (Shen et al., 2022). Interestingly, despite their A-type crystalline form, Seullomi accessions maintained higher SDS levels after cooking, suggesting that structural resistance may also involve favorable fine-chain amylopectin organization and other physicochemical features that merit further investigation.

Textural analysis supported these structural observations. Seullomi1 and Seullomi2 demonstrated moderate hardness and stickiness values comparable to the commercial variety Sindongjin, whereas Dodamssal exhibited significantly higher hardness and minimal stickiness, likely attributed to its high RS content and cooking-resistant starch configuration (**Figure 2**). As this study focused solely on instrumental texture parameters, a comprehensive sensory analysis including evaluations of aroma, flavor, and visual characteristics is needed for desirable eating quality for commercial potential.

The structural and textural data collectively indicate that Seullomi1 and Seullomi2 possess moderate to high amylose content, exhibit partial SDS retention after cooking, and demonstrate satisfactory physical properties, which make them strong candidates for low GI rice breeding. Conversely, while Dodamssal presents glycemic advantages, it may suffer from limited consumer appeal due to its firm and non-sticky texture. These findings underscore the importance of identifying rice accessions that effectively balance glycemic performance with sensory acceptability and highlight the need for further molecular profiling to elucidate the structural features that contribute to SDS persistence.

### 4.5 Implications for low GI rice breeding and future directions

The findings of this study provide critical insights into starch digestibility traits relevant to the development of low GI rice. Dodamssal demonstrated the lowest GI among the evaluated accessions, primarily driven by its very high amylose content and associated RS2 formation. However, its firm texture and extremely low stickiness may limit consumer preference. In contrast, Seullomi1 and Seullomi2 exhibited moderate-to-high amylose levels and significantly higher SDS retention following cooking, resulting in a lower postprandial glycemic response while maintaining more favorable textural attributes. This combination suggests that optimizing the proportion of amylose and SDS, rather than focusing exclusively on RS accumulation, may represent a practical strategy for breeding commercially acceptable low GI rice.

Importantly, the contribution of SDS to glycemic moderation should be interpreted as associative rather than mechanistically independent, since the current experimental design does not control for amylose background. While SDS retention appears advantageous, amylose content and starch molecular configuration remain the dominant regulatory factors affecting digestion rate. Thus, future breeding programs should prioritize the identification and selection of accessions that combine moderate amylose content, stable SDS retention after cooking, and acceptable texture characteristics. Integrating alleles associated with fine-chain amylopectin structure, starch crystallinity, and protein–starch interactions may enhance the breeding potential of these lines.

Advances in marker-assisted selection, starch functional genomics, and transcriptomic profiling could expedite the identification of candidate loci involved in shaping SDS maintenance and starch hierarchy. For Seullomi-based germplasm, further investigation into starch fine architecture (e.g., degree of polymerization distribution, crystalline–amorphous transition characteristics) may help refine trait selection strategies. Additionally, human clinical validation and comprehensive sensory evaluation, including aroma, taste, and visual quality, are required before proposing these accessions for commercial applications.

Overall, Seullomi1 and Seullomi2 represent promising prototypes for the development of functional rice varieties capable of moderating glycemic response while maintaining textural quality. Their refinement through strategic breeding and molecular dissection may enable the development of rice varieties that better respond to nutritional and consumer demands, supporting public health strategies aimed at reducing the burden of type 2 diabetes.

## 5 Conclusion

This study provides a comprehensive evaluation of the morphological, biochemical, structural, and glycemic properties of 16 Korean rice accessions intended for low GI rice development. Dodamssal exhibited the lowest GI among all accessions, primarily due to its very high amylose content and the associated formation of RS2, which limited starch gelatinization and reduced enzymatic accessibility. However, its firm texture and low stickiness may restrict consumer acceptance.

In contrast, Seullomi1 and Seullomi2 presented low-to-intermediate GI values despite having only moderate to high amylose levels. Their ability to retain SDS after cooking, together with acceptable hardness and stickiness, indicates that these accessions may offer a more balanced combination of nutritional benefits and edible quality. While SDS appears to play a complementary role in moderating glycemic response, its effect cannot be interpreted as independent from amylose-mediated structural contributions, and should therefore be considered associative rather than causal based on the current experimental design.

These findings highlight that effective breeding strategies for low GI rice should focus not solely on increasing RS but on achieving an optimal balance among amylose content, SDS retention, and favorable texture. Seullomi1 and Seullomi2 represent promising prototypes that align with consumer-oriented health objectives. Future work should prioritize genotype-controlled studies to determine the independent contribution of SDS, alongside detailed molecular investigation of starch architecture and comprehensive sensory evaluation. Human clinical trials will be essential to confirm long-term glycemic benefits. Consequently, these accessions provide valuable resources for breeding functional rice varieties that support metabolic health while meeting consumer quality expectations.

## Data Availability

The original contributions presented in the study are included in the article/supplementary material, further inquiries can be directed to the corresponding author/s.
